# Skeletal and Dentoalveolar Cephalometric Features of Anterior Open Bite among Yemeni Adults

**DOI:** 10.1155/2016/3147972

**Published:** 2016-04-12

**Authors:** Ammar Abdulkareem Daer, Amal Hussein Abuaffan

**Affiliations:** ^1^Department of Orthodontics, Faculty of Dentistry, Sana'a University, Sana'a, Yemen; ^2^Department of Orthodontics, Pedodontics and Preventive Dentistry, Faculty of Dentistry, University of Khartoum, Khartoum, Sudan

## Abstract

*Objective.* The aim of this study is to determine the cephalometric features for a sample of Yemeni adults with anterior open bite.* Material and Methods*. Lateral cephalometric radiographs were taken for 65 Yemeni university students (46 males and 19 females), 18–25 years old, with clinical anterior open bite (vertical overbite ≤ 0 mm) and no previous orthodontic treatment. The radiographs were manually traced; twelve angular, five linear measurements, and facial index were assessed, analyzed statistically, and compared to 194 Yemeni norms (89 males and 105 females) as well as cephalometric features of open bite subjects in previous studies.* Results.* Statistically significant differences were observed in skeletal and dental cephalometric values of Yemeni patients with anterior open bite when compared to Yemeni norms; mainly in the anteroposterior relation, the open bite individuals had higher significant value in SNA, SNB, and SNPg angles. In addition, a higher statistical significant difference was recorded in all variables of vertical relation when compared with norms. In contrast, NL-NSL angle revealed higher value among normal individuals. Dental variables among open bite individuals showed a greater degree of dental proclination, higher statistically significant value in $\underline{I}$-NA°, $\underline{I}$-NA mm and I-NB mm, and a lower significant value in U1-L1 in open bite group.* Conclusion.* Open bite Yemeni individual's skeletal and dentoalveolar variables significantly differ from Yemeni norms in the extent of the anteroposterior, vertical developmental pattern and dental relations.

## 1. Introduction

Anterior open bite is a major occlusal disorder in the vertical direction, which is often associated with defects in the anteroposterior direction. It is one of the most difficult malocclusions which constitutes a real challenge to orthodontics [1, 2]. It was defined by several authors as the absence of coverage between the incisors (upper and lower) when the posterior teeth are in occlusal position [1–4].

According to McNamara and Burdon, open bite was classified into and skeletal. Dental open bite is localized to the anterior teeth and the surrounding soft and hard tissues without presenting any skeletal defect in cephalometric radiograph, whereas, skeletal open bite shows vertical disharmony in cephalometric radiograph [3]. Additional features to McNamara's classification had been added: pseudo open bite (presence of incisor protrusion), infantile open bite (involves all teeth and molar) and iatrogenic (occur due to careless orthodontic treatment as a result of using inappropriate rapid palatal expansion) [4].

Numerous studies in previous literature describe the skeletal and dental characteristics of the anterior open bite and discuss the various etiological factors that had a role in creating an open bite among different population [5–10].

Cephalometric radiograph has been used for the diagnosis of orthodontic problems in vertical and anteroposterior direction. Authors revealed different findings and measurement for open bite cases by using different methods of cephalometric analysis [11, 12].

In 1952, in the cephalometric analysis proposed by Wylie and Johnson, the vertical proportions of the normal face were measured along the line nasion-menton. The anterior nasal spine (ANS) served as the point of separation between the upper and the lower face height [11]. Later in 1964, Horowitz and Thompson measured the face height, upper face height, and lower face height along the nasion-gnathion line in a group of untreated normal postadolescent males and females [12].

Klocke et al. evaluated the craniofacial growth changes in a longitudinal study for two groups, 14 children (5 years old) in each, one group with anterior open bite and the other control group. Cephalometric measurements were analyzed at the ages of 5, 9, and 12 years [13].

Numerous studies concerning the cephalometric features of anterior open bite have given a vast amount of information in various parts of the world. Data about Yemeni open bite patients regarding skeletal and dentoalveolar features are rare. Therefore, the present study is designed to determine the skeletal and dental cephalometric features of anterior open bite among Yemeni adults in comparison to Yamani norms.

## 2. Materials and Methods

First, the ethical approval was obtained from the research committee of Sana'a University, Science and Technology University, and Dar Al-Salam University, to conduct this study. The aims of the study had been explained to all the students in the lecture room. Initial visual screening was carried out and the ones who had anterior open bite were registered and later on called and asked for further clinical examination at the dental clinic.

The total number of the students in the three faculties was 1585; out of these, 65 students had anterior open bite (19 females and 46 males) and fulfill the inclusion criteria of the current study: Yemeni nationality with no previous history of orthodontic or prosthodontic treatment, full permanent dentition and the vertical overbite ≤ 0 mm when teeth are in centric occlusion, and no craniofacial deformities. The normal cephalometric radiograph for Yemeni population had been studied and analyzed from one hundred ninety-four students (89 males and 105 females) who had normal occlusion and fulfill the inclusion criteria of the current study: Yemeni nationality with normal occlusion and balanced facial profile, full permanent dentition (except for the third molars), class I molar, incisor and canine relationship, normal overjet and overbite, normal transversal occlusion, and well aligned or crowded teeth not more than 2 mm; and no previous history of orthodontic treatment was registered.

Cephalograms were taken from each student after the consent form had been signed. Lead apron had been worn for protection and well trained operator takes the lateral, while the student head was in natural position and the teeth were closed in centric occlusion, the lips slightly closed and the right side of the subject face the x-ray source [14] .

**Figure 1:**
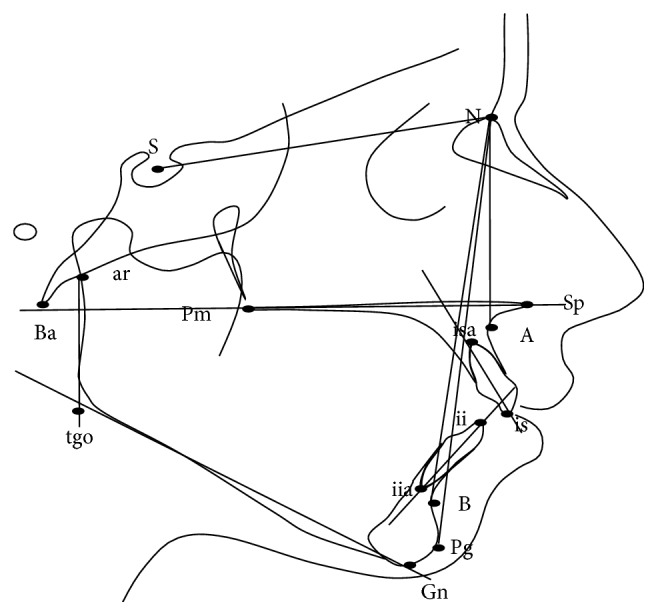
Cephalometric lines and angles.

The cephalograms were traced and measured by the main investigator. Five linear and twelve angular measurements were analyzed on each radiograph (Figure 1).

### 2.1. Statistical Analysis

Data were collected, summarized, cleaned, and coded. All statistical analyses were performed with the Statistical Package for Social Sciences (SPSS) program (version 20). For each variable, the arithmetic mean and standard deviation were calculated. Chi-square test was used. *P* value of less than 0.05 was considered as significant.

### 2.2. Reliability of the Measurements

The intraexaminer reliability test was carried out for error testing. Fifteen percent of the cephalograms were randomly selected and retraced in two-week interval by the same investigator; the first and second measurements were compared using paired Student's *t*-test.

## 3. Results: Reliability and Gender Differences

### 3.1. Reliability of the Measurements

A strong correlation was found between the first and second reading (Table 1).

A total of 65 Yemeni university students, 46 males and 19 females, were clinically diagnosed with anterior open bite. Cephalometric radiographs were traced and analyzed for the skeletal and dental features of the anterior open bite.

Table 2 showed the sagittal relationship; statistically significant differences were noted in SNA and SNPg angles, whereas, SNB, ANB and SNBa angles showed no significant difference between genders. Moreover, all angles measurements showed greater value in females than males except for SNBa angle.

According to Table 3, the vertical inclination and ML-NL and ML-NSL angles together with the upper and lower facial height measurement showed statistically significant differences between genders. In contrast, the facial index showed no significant difference.

In the dental measurements, only the upper incisor to NA (angle and line) showed statistically significant differences, whereas no significant differences between genders were observed in the other dental measurement values (Table 4).

## 4. Results: Open Bite Relative to Normal Occlusion (Yemeni)

### 4.1. Skeletal Relationships

#### 4.1.1. Anteroposterior

In the current study, all the skeletal variables showed significant differences between open bite and norms except for NSBa (Table 5) [15]. For SNA and SNB, the open bite group had lower value than the norms. Concerning SNBa angle, the Yemeni open bite had a higher value than that of norms.

#### 4.1.2. Vertical Inclination

In the present study, ML-NL angle showed higher value in open bite than Yemeni norms and the opposite was seen with NL-NSL angle which is higher in norms (Table 6) [15], whereas ML-NSL angle was less in value among the Yemeni norms and the Gn-tgo-Ar is higher in Yemeni open bite than in norms.

#### 4.1.3. Face Height

In the current study, the upper facial height (N-Sp′) was significantly higher in open bite Yemenis than in norms (Table 7).

For lower facial height, the Yemeni open bite had a significantly higher value than norms. Concerning the facial index, it was found that the Yemeni norms had significantly higher value than that of open bite.

#### 4.1.4. Dental Variables

In this study, the Yemeni open bite adults had greater bimaxillary proclination of the incisors, indicated by the smaller interincisal angle than norms which is statistically significant (Table 8).

The angles between the upper central incisor to the NA line and lower central incisor to the NB line showed a higher value in open bite Yemeni group than that of norms.

The liner measurement of $\underline{I}$-NA had a higher value in Yemeni open bite group than that of norms, while the liner measurement of $\bar{I}$-NB is found to be higher in open bite Yemenis than in norms.

## 5. Conclusion

- This was a cross-sectional study aimed at evaluating the skeletal and dental cephalometric features of 65 Yemeni university students (19 female and 46 male) aged 18–25 years with anterior open bite. Statistically significant differences were found in skeletal and dental relationships.
- From the results obtained among different population and Yamani results, it can be concluded that the Yemeni open bite adults had different skeletal and dentoalveolar cephalometric measurement when compared to other populations. These differences can be attributed partially to ethnic background, sample size and the genetic factors.
- The current study proved the results of previous studies among different population that skeletal and dental features had significant roles in the etiology of anterior open bite.

## Figures and Tables

**Table 1:** Reliability of measurements using reliability Student's *t*-test.

Variable	EM	*z* value	*P* value	Decision
SNA°	0.360	1.00	0.33	Ns
SNB°	0.234	0.00	1.00	Ns
ANB°	0.396	−1.36	0.19	Ns
SNPg°	0.308	1.44	0.16	Ns
NSBa°	0.349	0.57	0.57	Ns
ML-NL°	0.065	0.00	1.00	Ns
NL-NSL°	0.088	−1.00	0.33	Ns
ML-NSL°	0.134	−1.00	0.33	Ns
Gn-tgo-Ar°	0.126	1.00	0.33	Ns
N-Sp′ mm	0.275	−1.00	0.33	Ns
Sp′-Gn mm	0.053	0.00	1.00	Ns
Pg-NB mm	0.219	−1.00	0.33	Ns
Nod. angle	0.190	−0.57	0.57	Ns
I-I°	0.233	0.57	0.57	Ns
I-NA°	0.243	−0.57	0.57	Ns
I-NB°	0.427	1.00	0.33	Ns
I-NA mm	0.024	1.00	0.33	Ns
I-NB mm	0.247	0.00	1.00	Ns

**Table 2:** The skeletal anteroposterior variables for Yemeni adults with anterior open bite.

Variables	Male, 46				Female, 19				*P* value
	Mean	Max.	Min.	SD	Mean	Max.	Min.	SD	
SNA°	77.23	87	71	4.05	79.59	85	76	2.55	0.022^*∗*^
SNB°	73.57	84.3	68	4.01	75.03	79.8	69.5	2.71	0.151
ANB°	3.68	7	−2	2.27	4.56	10	1	2.18	0.155
SNPg°	73.8	85	67	4.35	75.91	81	71	2.68	0.021^*∗*^
SNBa°	134.14	143	123	5.55	131.26	142	120	5.34	0.059

^*∗*^ *P* < 0.05 is significant.

**Table 3:** The skeletal vertical variables for Yemeni adults with anterior open bite.

Variables	Male, 46				Female, 19				*P* value
	Mean	Max.	Min.	SD	Mean	Max.	Min.	SD	
ML-NL°	37.53	50	23.5	8.02	32.69	43.5	23	5.31	0.006^*∗*^
NL-NSL°	7.8	12	2	3.19	9.26	16	4	2.86	0.089
ML-NSL°	45.2	56	31	6.96	41.45	54	32.5	5.91	0.044^*∗*^
Gn-tgo-Ar°	128.11	144	116	8.09	126.54	141	116.5	6.6	0.458
N-Sp′ mm	70.86	77	53	5.04	61.02	71.3	52	6.5	0.000^*∗*^
Sp′-Gn mm	99.04	119	78.8	10.93	85.11	98.28	64.57	12.25	0.000^*∗*^
N-Sp/Sp-Gn	0.7200	0.82	0.62	0.0567	0.7242	0.83	0.56	0.0728	0.804

^*∗*^ *P* < 0.05 is significant.

**Table 4:** Distribution of dental variables among Yemeni adults with anterior open bite.

Variables	Male, 46				Female, 19				*P* value
	Mean	Max.	Min.	SD	Mean	Max.	Min.	SD	
1-1°	120.74	133.5	110	5.38	121.13	137.5	105.5	8.94	0.862
I-NA°	27.4	35	17.5	4.72	23.79	35	15	5.58	0.011^*∗*^
*Ī*-NB°	28.01	35	21	4.5	29.66	43	20	6.02	0.230
I-NA mm	7.21	11.5	3.5	2.08	5.48	12.76	174	2.99	0.010^*∗*^
*Ī*-NB mm	9.15	13.36	5.68	2.19	8.48	16.95	4.16	3.8	0.477

^*∗*^ *P* < 0.05 is significant.

**Table 5:** The skeletal anteroposterior variables for normal and open bite Yemeni adults.

Variables	Open bite (*N* 65)		Norm bite (*N* 194)		*P* value
	Mean	Std.	Mean	Std.	
SNA°	77.9	3.81	80.86	2.54	0.000^*∗*^
SNB°	73.99	3.72	77.89	2.52	0.000^*∗*^
ANB°	3.94	2.26	2.97	1.35	0.002^*∗*^
SNPg°	74.4	4.03	78.67	2.6	0.000^*∗*^
NSBa°	133.3	5.61	131.71	6.05	0.063

^*∗*^ *P* < 0.05 is significant.

**Table 6:** The skeletal vertical variables for open bite and norm Yemeni adults.

Variables	Open bite (*N* 65)		Norm bite (*N* 194)		*P* value
	Mean	Std.	Mean	Std.	
ML-NL°	36.1	7.62	21.36	5.43	0.000^*∗*^
NL-NSL°	8.22	3.15	9.87	3.75	0.002^*∗*^
ML-NSL°	44.1	6.84	31.23	5.53	0.000^*∗*^
Gn-tgo-Ar°	127.65	7.67	119.3	7.3	0.000^*∗*^

^*∗*^ *P* < 0.05 is significant.

**Table 7:** The facial height variable for open bite and norm Yemeni adults.

Variables	Open bite (*N* 65)		Norm bite (*N* 194)		*P* value
	Mean	Std.	Mean	Std.	
N-Sp′ mm	67.98	7.08	60.03	6.48	0.000^*∗*^
Sp′-Gn mm	94.97	12.92	73.29	8.82	0.000^*∗*^
N-Sp′/SP′-Gn × 100	0.72	0.06	0.82	0.07	0.000^*∗*^

^*∗*^ *P* < 0.05 is significant.

**Table 8:** Distribution of the dental variable for open bite and norm Yemeni adults.

Variables	Open bite (*N* 65)		Norm bite (*N* 194)		*P* value
	Mean	Std.	Mean	Std.	
1-1°	120.85	6.55	126.65	7.19	0.000^*∗*^
I-NA°	26.3	5.21	21.57	4.96	0.000^*∗*^
*Ī*-NB°	28.49	5	27.96	4.5	0.419
I-NA mm	6.7	2.48	4.6	1.78	0.000^*∗*^
*Ī*-NB mm	8.95	2.74	6.26	2.14	0.000^*∗*^

^*∗*^ *P* < 0.05 is significant.
